# The risk factors of local recurrence and distant metastasis on pT1/T2N0 mid-low rectal cancer after total mesorectal excision

**DOI:** 10.1186/s12957-021-02223-4

**Published:** 2021-04-13

**Authors:** I-Li Lai, Jeng-Fu You, Yih-Jong Chern, Wen-Sy Tsai, Jy-Ming Chiang, Pao-Shiu Hsieh, Hsin-Yuan Hung, Yu-Jen Hsu

**Affiliations:** Division of Colon and Rectal Surgery, Chang Gung Memorial Hospital, Linkou, No.5, Fu-Hsing Street, Guei-Shan, Tao-Yuan, Taiwan

**Keywords:** Rectal cancer, Local recurrence, Distant metastasis, Radical resection, Total mesorectal excision

## Abstract

**Background:**

Radical resection is associated with good prognosis among patients with cT1/T2Nx rectal cancer. However, still some of the patients experienced cancer recurrence following radical resection. This study tried to identify the postoperative risk factors of local recurrence and distant metastasis separately.

**Methods:**

This retrospective, single-center study comprised of 279 consecutive patients from Linkou branch of Chang Gung Memorial Hospital in 2005–2016 with rectal adenocarcinoma, pT1/T2N0M0 at distance from anal verge ≤ 8cm, who received curative radical resection.

**Results:**

The study included 279 patients with pT1/pT2N0 mid-low rectal cancer with median follow-up of 73.5 months. Nineteen (6.8%) patients had disease recurrence in total. Nine (3.2%) of them had local recurrence, and fourteen (5.0%) of them had distant metastasis. Distal resection margin < 0.9 (cm) (hazard ratio = 4.9, *p* = 0.050) was the risk factor of local recurrence. Preoperative carcinoembryonic antigen (CEA) ≥ 5 ng/mL (hazard ratio = 9.3, *p* = 0.0003), lymph node yield (LNY) < 14 (hazard ratio = 5.0, *p* = 0.006), and distal resection margin < 1.4cm (hazard ratio = 4.0, *p* = 0.035) were the risk factors of distant metastasis.

**Conclusion:**

For patients with pT1/pT2N0 mid-low rectal cancer, current multidisciplinary treatment brings acceptable survival outcome. Insufficient distal resection margin attracted the awareness of risk factors for local recurrence and distant metastasis as a foundation for future research.

**Supplementary Information:**

The online version contains supplementary material available at 10.1186/s12957-021-02223-4.

## Background

Transabdominal radical resection without neoadjuvant therapy is recommended for patients with rectal cancer at clinical T1/T2 and negative N stage [1], and this sphincter-saving surgery with total mesorectal excision (TME) has been associated with high survival rates and low recurrence rate [2].

On the other hand, a growing number of patients with clinical T1/T2 tumors have undergone local excision (LE) which has improved their quality of life. However, concerns remain surrounding treatment, and though quality of life has improved, patients may still be at higher risk for disease recurrence [3, 4]. Radical resection generally guarantees disease-free survival at the expense of quality of life. Still, some patients with radical resection experience cancer recurrence which can be very frustrating and discouraging for both the patients and surgeons.

Previous studies reporting on rates of local recurrence (LR) and distant metastasis (DM) in patients with rectal cancer have not been consistent and owing to the limited data available, and the clear recommendations for preventing rectal cancer recurrence have not been established well. By knowing the risk factors, improvements in surgical planning and follow-up strategies may help improve cancer-free survival. Therefore, our study aimed to identify the risk factors for postoperative LR and DM in those with early-stage rectal cancer.

## Methods

**Fig. 1:**
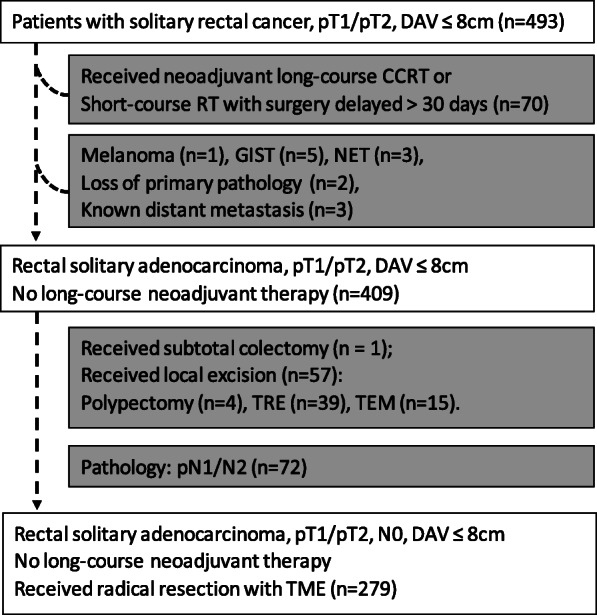
Patient selection of this study: patients with mid and low rectal cancer with lesions ≤8 cm away from the anal verge were included. Patients who were administered long-term (more than 30 days) neoadjuvant chemoradiotherapy or received radical resection after waiting for more than 30 days after receiving short-term (5 days) radiotherapy were excluded from this study due to the possibility of tumor regression. Other histologic types other than adenocarcinoma were listed and excluded. Patients who received subtotal colectomy due to familial polyposis, local excision including four who underwent transrectal polypectomies, 39 who had transanal excisions, and 15 who underwent transanal endoscopic microsurgeries were excluded. In the last step, we moved the patients who had positive N stage to supplement information

Data was retrieved from medical records of Chang Gung Memorial Hospital (CGMH) between 2005 and 2016, from 493 adult patients who had pT1/T2 rectal cancer; the data was finally collected from 279 patients with solitary, localized, resectable pT1/T2, N0 rectal adenocarcinomas with a distance from anal verge (DAV) ≤ 8 cm (Fig. 1). This study was approved by Institutional Review Board of CGMH with number 202000644B0. Rectal sonography, pelvic MRI, and PET-CT were performed for clinical staging at cT1/T2. All patients received chest to pelvis CT to assess for preoperative occult metastasis. If the patients received neoadjuvant therapy, only the patients who received short-course radiotherapy (RT) with 500 cGy × 5 days and underwent TME within 7 days were included. All patients received radical TME with curative intent. This procedure can be performed as an open method, laparoscopically assisted, or as a robotic surgery. All specimens were examined carefully by a well-trained pathologist with precise pT1/T2 (Table 1).

**Table 1:** Patient characteristics for pT1/T2, N0

Variable	279 patients (% or [Q1 − Q3] †)	
	No recurrence (*n*=260, %)	Recurrence (*n*=19, %)
Age	63.8± 12.4	58.4 ± 11.0
BMI (kg/m^2^)	24.1± 3.2	24.8 ± 3.4
Male gender	141 (54.2)	12 (63.2)
Family cancer history	79 (30.4)	8 (42.1)
Preoperative CEA (ng/mL)	1.8 [1.1–2.7] ^†^	2.3 [1.5–6.7] ^†^
Preoperative CEA ≥ 5*	30 (11.5)	7 (36.8)
Preoperative hemoglobin (g/dL)	12.8 ± 2.0	13.8 ± 1.8
Preoperative albumin (g/dL)	4.23 ± 0.40	4.32 ± 0.37
**Distance from anal verge** (cm)*	5.9 ± 1.7	4.9 ± 1.9
Distance from anal verge ≤ 5	108 (41.5)	12 (63.2)
Operation type		
Low anterior resection	241 (92.7)	17 (89.5)
Abdomino-perineal resection	17 (6.5)	2 (10.5)
Hartmann’s procedure	2 (0.8)	0
Neo-adjuvant radiotherapy ^‡^	41 (15.8)	6 (31.6)
Adjuvant therapy	4 (1.5)	0
Chemotherapy	3 (1.2)	0
CRT	1 (0.4)	0
PeriOP colostomy/ileostomy	149 (57.3)	15 (78.9)
PostOP complication/morbidity	68 (26.2)	3 (15.8)
Early	43 (16.5)	3 (15.8)
Late	37 (14.2)	1 (5.3)
Resection margin (cm)	1.5 [0.8–2.2] ^†^	0.8 [0.5–1.7] ^†^
Resection margin < 0.9 *	78 (30.0)	10 (52.6)
Resection margin < 1.4 *	118 (45.4)	14 (73.7)
Tumor diameter (cm)	3.0 ± 1.4	3.3 ± 1.1
Tumor diameter ≥ 2.7	137 (52.7)	13 (72.2)
T stage		
T1	98 (37.7)	5 (26.3)
T2	162 (62.3)	14 (73.7)
Lymph node yield	20 [14–28] ^†^	16 [11–33] ^†^
Lymph node yield ≥ 14*	207 (79.6)	11 (57.9)
Lymphovascular invasion	12 (4.6)	1 (5.3)
Perineural invasion	13 (5.0)	0
Differentiation		
Poor	4 (1.5)	1 (5.3)
Moderate	195 (75.0)	15 (78.9)
Well	61 (23.5)	3 (15.8)
Follow-up (month)	73.5 [48–108] ^†^	
Total follow-up length	79.6 [51–109] ^†^	64.9 [54–102] ^†^
Time to local recurrence		25.6 [13.7–38.8] ^†^
Time to distant metastasis		31.4 [12.9–59.2] ^†^

*BMI* body mass index, *CEA* carcinoembryonic antigen, *CRT* chemoradiotherapy

*p value < 0.05

^†^Median [25 percentile–75 percentile]

^‡^Short-course radiotherapy 500cGy*5days

Following discharge, all patients returned to the clinic following a 7–10-day period for assessment. Patients were advised to return to the clinic for carcinoembryonic antigen (CEA) evaluations and chest x-rays every 3 months. As part of the follow-up evaluations, patients also underwent computed tomography (CT) and colonoscopy annually for the first 3 years following the surgery. LR was defined as intrapelvic recurrence to the area of anastomosis, presacral space, anterior side of the rectum, to organs with adhesions found in close proximity, internal iliac nodes, and lateral pelvic wall. DM was defined as recurrence outside the pelvic cavity detected after at least 6 months following curative resection.

We used receiver operating characteristic curve (ROC curve), which provided area under the curve (AUC), to determine the cutoff point for distance from anal verge (DAV), the lymph node yield (LNY), tumor diameter, and distal resection margin (DRM). After the cutoff points were identified, we examined the risk factors including family cancer history, sex, high CEA level (≥ 5 ng/mL), rate of postoperative morbidity (early and late), preoperative radiotherapy, T stage, lymphovascular invasion (LVI), perineural invasion (PNI), and tumor cell differentiation with Kaplan-Meier survival analysis. If the “*p* value < 0.1” was observed from Log rank test, then, we applied the risk factor into the COX regression model. A univariate COX regression model was applied followed by multivariate COX regression model in backward stepwise (Wald) that was used to provide an estimate of the hazard ratio (HR) and its confidence interval (CI) for investigating the association between the survival time of patients and one or more predictor variables/factors.

## Results

Overall, 279 patients with pT1/pT2 mid-low rectal cancer were included in the analysis. The median follow-up period was 73.5 months. Overall, 19 (6.8%) patients had disease recurrence. Nine (3.2%) had LR, and 14 (5.0%) had DM. The median interval of time to recurrence was 25.6 months for LR and 31.4 months for DM. Three- and 5-year disease-free survival were 90% and 86%, respectively, while the 3- and 5-year cumulative recurrence rates were 4% and 6%, respectively.

**Fig. 2:**
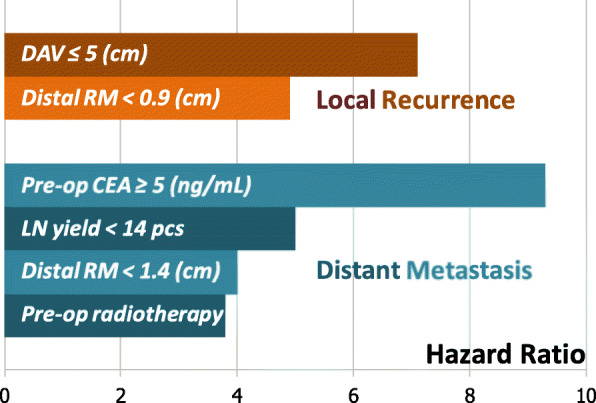
Hazard ratio for local recurrence and distant metastasis

After univariable COX regression, we selected these factors below for multivariable COX regression. CEA ≥ 5 with HR = 9.3 (95% CI 2.79–30.76, *p* = 0.0003), LNY < 14 with HR = 5.0 (95% CI 1.57–15.63, *p* = 0.006), DRM < 1.4 (cm) with HR = 4.0 (95% CI 1.10–14.41, *p* = 0.035), and preoperative radiotherapy with HR = 3.8 (95% CI 1.27–11.13, *p* = 0.035) were risk factors for DM. DRM < 0.9 (cm) with HR = 4.9 (95% CI 1.00–24.42, *p* = 0.050) and DAV ≤ 5 (cm) with HR = 7.1 (95% CI 0.86–59.19, *p* = 0.068) were risk factors for LR with borderline significance. All adjusted parameters, HR along with 95% CI and *p* value, are listed in Table 2 and Fig. 2.

**Table 2:** Risk factors for local recurrence and distant metastasis in Cox regression model

Variable	Univariable		Multivariable	
	Hazard ratio (95% CI for Exp(B))	*p* value	Hazard ratio (95% CI for Exp(B))	*p* value
**Local recurrence** ^†^				
Distal resection margin < 0.9 (cm)	7.7 (1.60–37.17)	0.011*	4.9 (1.00–24.42)	0.050
Distance from anal verge ≤ 5 (cm)	11.0 (1.37–87.89)	0.024*	7.1 (0.86–59.19)	0.068
Family cancer history	2.9 (0.78–10.88)	0.111		
Preoperative radiotherapy	2.0 (0.24–15.93)	0.533		
T stage	1.1 (0.28–4.50)	0.878		
**Distant metastasis**^‡^				
CEA ≥ 5 (ng/mL)	6.1 (2.06–18.26)	0.001*	9.3 (2.79–30.76)	0.0003*
Lymph node yield < 14	3.5 (1.22–9.93)	0.020*	5.0 (1.57–15.63)	0.006*
Distal resection margin < 1.4 (cm)	4.4 (1.23–15.91)	0.023*	4.0 (1.10–14.41)	0.035*
Tumor diameter ≥ 2.7 (cm)	3.2 (0.89–11.50)	0.074		
Preoperative radiotherapy	3.4 (1.16–9.86)	0.025*	3.8 (1.27–11.13)	0.016*
T stage	1.4 (0.44–4.47)	0.575		

*CEA* carcinoembryonic antigen

*p value < 0.05

^†^Adjusted parameters (local recurrence): distal resection margin, distance from anal verge, family cancer history, preoperative radiotherapy, and T stage

^‡^Adjusted parameters (distant metastasis): CEA, distal resection margin, lymph node yield, tumor diameter, pre-operative radiotherapy, and T stage

Overall, 44% patients with LR were first evaluated by digital exam and subsequently diagnosed, while 71% of patients with DM were detected first by CEA elevation. Three (33%) patients with endoluminal LR and six (66%) with presacral or perirectal recurrence were identified in LR group. Eight (57%) patients with lung metastases and seven (50%) with liver metastases were identified in the DM group. There were 9 local recurrences and 14 distant metastases remaining from 19 patients. In these 4 patients who had both local recurrence and distant metastasis, two of them were detected at the same time. For the other two patients, one of them was detected local recurrence at postoperative 3 years, and then, CEA elevation came with the detection of lung metastasis 8 months later; the other was detected local recurrence at postoperative 15 months, and then, CEA elevation came with the detection of bone metastasis 2.5 years later.

## Discussion

Currently, the published data on recurrence rates for patients with pT1/T2 mid-low rectal cancer is very limited. Pre-treatment CEA elevation, T2 stage, tumor distance from anal verge, close distal resection margin, lymphovascular invasion, perineural invasion, young age, male gender, ulcerative gross appearance (rather than polypoid appearance), and anastomotic leakage have been reported for risk factors of tumor recurrence, time to recurrence, and/or the recurrence patterns [5–10]. However, there was no consensus result, and sometimes, controversy existed. Some of the studies focused on transanal endoscopic surgery, which might have different results from those who received TME; some of the studies excluded patients who received any type of neoadjuvant therapy, which may generate another type of selection bias. For real-world data, some patients may receive neoadjuvant therapy due to clinically suspicious advanced T stage or possible N+ stage. After the specimen is examined, patients at pathological stage III were recommended to adjuvant chemotherapy and excluded from this research (Supplementary table 1).

### Local recurrence

However, the existing reports suggest that once surgical treatment is performed and R0 resection is confirmed, good outcomes can be expected [11]. The most common sites for locoregional recurrence are generally the area around the anastomotic site, anterior side of the rectum, and the presacral site [12]. For those with mid-low rectal cancer, failure to achieve successful TME or receive preoperative RT may cause LR [13]. Lower DAV increases the difficulty of the surgery, and thus is thought to have a negative impact on survival. One recent prospective study reported a higher proportion of patients with positive resection margins in those with rectal cancer <5 cm DAV [6]. DAV has also been found to impact metastatic spread to the liver and lungs, a finding that was consistent with our data showing that those with mid-low rectal cancer had higher rates of lung metastasis [10].

In the US and European societies, perioperative RT is considered an acceptable adjuvant treatment for controlling LR. One Dutch TME trial reported lower 5-year LR rates in a TME + RT group than that for a TME only group (4.6% vs 11%) [14]. Another recent study reported no benefits associated with long-term neoadjuvant chemoradiotherapy (CRT) in terms of reduced early-stage rectal cancer recurrence [15]. In our study, one of the 47 patients who received short-course RT following TME had LR. The policy of preoperative radiotherapy in our hospital usually suggests for clinical T3 stage and above or positive N stage. However, it was flexible for mid-low rectal cancers in some circumstances. There was no significant difference for LR between RT and non-RT groups, but patients in the RT group tended to develop DM, and this might be related to patient selection policy. Patients in the RT group had higher perioperative colostomy/ileostomy rate; this group had higher proportion of T2 tumor and lower proportion of well-differentiated tumor (supplementary table 2).

### Carcinoembryonic antigen (CEA)

CEA is a protein produced during prenatal development that decreases to very low or undetectable levels following delivery. In current practice, CEA is mostly utilized to complete preoperative evaluations and to assess patients for occult recurrence of colorectal cancer on follow-up. In recent studies, high pretreatment CEA was regarded as a poor prognostic factor for colorectal cancer after curative surgery [7, 16]. In a retrospective study that included 16,659 patients, elevated pre-operative CEA levels predicted poor prognosis much more accurately in pT1 patients who were considered to have a better prognosis according to the TNM system [17]. In our study, 37 patients had preoperative CEA elevation. Seven (18.9%) of them had two LRs, and six had DM (one of them had both LR and DM) when evaluated during the postoperative follow-up. Preoperative CEA elevation was considered to be a poor prognostic factor in our study.

### Lymph node yield (LNY)

The presence of metastatic LNs identified by pathological examination indicates systemic tumor spread and is therefore the major determinant for adjuvant therapy. There is a current consensus that at least 12 lymph nodes (LNs) should be yielded when obtaining the surgical specimen in order to conduct an appropriate pathological examination; appropriate LNY can help to stage colorectal cancer more precisely. Inappropriate LNY may lead to underreporting, and thus result in higher recurrence rates and poorer survival [18].

LNY number is possibly affected by factors such as age, gender, tumor size, location, T stage, N stage, preoperative CRT, tumor regression grade, or the pathologic investigation [19, 20]. A few recent large-scale retrospective studies reported survival benefits with LNY ≥ 12 in those with colorectal cancer [20, 21]. However, rectal cancer is thought to be more difficult than colon cancer in achieving a LNY ≥ 12 [22]. In our study, 279 patients had a median LNY of 20. Overall, 244 of 279 (87.5%) patients had a LNY ≥ 12.

Recently, one large SEER database retrospective study based on 154,208 patients with colon cancer found that LNY did not have a unique, strong threshold for assessing survival (i.e., 12 lymph nodes) [23]. Interestingly, the study reported that patients without LN metastasis had a lower risk of death for each LN examined up to approximately 25 LNs. With a higher LNY, oversights made in staging due to false-negative N stages might decrease. Some studies reported survival benefits with a LNY ≥ 14 or more. The effect on an adequate LNY might bring survival benefits even for those at a pN0 stage [24]. This suggests that the survival benefits associated with increasing LNY may not be completely associated with N stage. A possible explanation is that an increased number of negative lymph nodes are associated with a higher immune response and longer survival [25]. In our study, we used ROC curve and identified that LNY = 13.5 had the largest AUC for 280 pN0 patients. For our analysis, those with LNY ≥ 14 had better outcomes in distant-metastasis-free survival (*p* = 0.013) and disease-free survival (*p* = 0.047).

### Distal resection margin

In our study, DRM was found to be a significant risk factor for both LR (<0.9 cm) and DM (<1.4 cm). Retained intramucosal cancer cells can potentially increase the risk of resection site recurrence, and migration of cancer to the perirectal tissue may lead to locoregional recurrence in the pelvic cavity. In addition, insufficient DRM is associated with a higher risk of LR [26]. Though the 1-cm rule is still controversial in some studies, especially for patients who undergo preoperative RT [12, 27], a DRM 1 to 2 cm is acceptable according to the current NCCN guidelines [28]. Some studies regarding transanal TME revealed that with appropriate DRM, short-term and long-term oncological outcomes improved for those with mid-low rectal cancers [29–31], and so forth, transanal TME may provide better outcome from preventing DM in correlation to our findings.

### Miscellaneous

Age, postoperative complication, LVI, and T stage may be risk factors for LR and/or DM. A meta-analysis that included five prospective cohort and six retrospective cohort studies reported that anastomotic leakage after radical resection of rectal cancer adversely impacted cancer-specific mortality and LR [5]. Age younger than 63 and DAV ≤ 5 cm were reported to have a higher chance of early DMs in a recent study [16]. In addition, LVI was reported to be a risk factor of DM in some studies [7]. However, this factor was not significant in our study. Those with advanced T stage tend to have poor prognoses and higher risk of disease recurrence; however, our study did not reveal the difference in impact between T1 and T2 stage on disease-free or recurrence-free survival. The possible explanation was the patient selection policy which may encourage pT2 for radiotherapy because of the inaccurate preoperative clinical staging.

### Limitations

Our study was limited by its retrospective design, small case number, and experience from a single tertiary center. All patients were treated by the same colorectal team, including surgeons with similar training background and surgical concepts, and this may have led to similar preferences among the surgeons, which may have resulted in bias. The selection criteria for preoperative radiotherapy were a confounding factor. Even though the study had a long-term follow-up period, the evolution of surgical techniques could not be evaluated.

## Conclusion

For patients with pT1/pT2N0 mid-low rectal cancer, multidisciplinary management that includes awareness of risk factors for local recurrence and distant metastasis is needed for treatment and to improve survival outcomes. Our study identified distal resection margin < 0.9 (cm) to be the main risk factor of local recurrence, while CEA ≥ 5 (ng/mL), lymph node yield < 14, and distal resection margin < 1.4 (cm) were risk factors for distant metastasis. For achieving more sufficient distal resection margin by the surgical planning and evolution of technique and devices, we hope that the current study can lay a foundation to improve survival outcomes in the future.

## Supplementary Information

**Additional file 1: Supplementary table 1** Patient characteristics

**Additional file 2: Supplementary table 2** Patient characteristics for non-RT vs. RT

## Data Availability

The detailed patients’ databases generated and analyzed during this study are not publicly available due to appropriate protection of patients’ personal information but are available from the corresponding author on reasonable request.

## Abbreviations

CEA: Carcinoembryonic antigen
CGMH: Chang Gung Memorial Hospital
CI: Confidence interval
CRT: Chemoradiotherapy
CT: Computed tomography
DAV: Distance from anal verge
DM: Distant metastasis
DRM: Distal resection margin
HR: Hazard ratio
LAR: Low anterior resection
LE: Local excision
LNY: Lymph node yield
LR: Local recurrence
LVI: Lymphovascular invasion
RT: Radiotherapy
TME: Total mesorectal excision
