# Supplementary material for: The risk factors of local recurrence and distant metastasis on pT1/T2N0 mid-low rectal cancer after total mesorectal excision
Source: World J Surg Oncol. 2021 Apr 13;19:116. doi: 10.1186/s12957-021-02223-4 (PMC8045195; doi:10.1186/s12957-021-02223-4)
Supplement: Supplementary file 1 — Additional file 1: Supplementary table 1 Patient characteristics [file 12957_2021_2223_MOESM1_ESM.docx]

**Supplementary table 1** Patient characteristics

| Variable | All 352 patients (% or [Q1 - Q3] †) | | |
| --- | --- | --- | --- |
|  | No Recurrence  (n=324, %) | | Recurrence  (n=28, %) |
| **Age** * | 63.7± 12.4 | 58.9 ± 11.6 | |
| **Age < 55** * | 79 (24.4) | 12 (42.9) | |
| BMI (kg/m2) | 24.1± 3.4 | 24.6 ± 3.4 | |
| Male Gender | 174 (53.7) | 15 (53.6) | |
| Family cancer history | 107 (33.0) | 13 (46.4) | |
| **Pre-operative CEA (ng/mL)** | 1.8 [1.1- 2.8] † | 2.2 [1.3 - 5.1] † | |
| **Pre-operative CEA ≥ 5** * | 34 (10.5) | 7 (25.0) | |
| Pre-operative Hemoglobin (g/dL) | 13.0 ± 1.9 | 13.5 ± 1.8 | |
| Pre-operative Albumin (g/dL) | 4.26 ± 0.39 | 4.25 ± 0.40 | |
| **Distance from anal verge (cm)** * | 5.9 ± 1.8 | 5.0 ± 1.9 | |
| **Distance from anal verge ≤ 5** * | 135 (41.7) | 18 (64.3) | |
| Operation type |  |  | |
| Low anterior resection | 297 (91.7) | 23 (82.1) | |
| Hartmann’s procedure | 3 (0.9) | 0 | |
| Abdomino-perineal resection | 23 (7.1) | 4 (14.3) | |
| Subtotal colectomy | 1 (0.3) | 1 (3.6) | |
| Neo-adjuvant radiotherapy ‡ | 48 (14.8) | 8 (28.6) | |
| Adjuvant therapy | 53 (16.4) | 7 (25.0) | |
| Chemotherapy | 51 (15.7) | 7 (25.0) | |
| CRT | 2 (0.7) | 0 | |
| Peri-OP colostomy/ileostomy | 183 (56.5) | 19 (67.9) | |
| Post-OP complication/morbidity | 86 (26.5) | 9 (32.1) | |
| Early | 57 (17.6) | 8 (28.6) | |
| Late | 43 (13.3) | 2 (7.1) | |
| **Resection margin** (cm) * | 1.5 [0.8– 2.2] † | 1.3 [0.5 – 1.7] † | |
| **Resection margin < 1.5 (cm)** * | 152 (46.9) | 19 (67.9) | |
| Tumor diameter (cm) | 2.8 [2.0 – 3.8] † | 3.0 [2.1 – 4.0] † | |
| Tumor diameter (cm) ≥ 3 (cm) | 151 (46.6) | 16 (59.3) | |
| T stage |  |  | |
| T1 | 111 (34.3) | 8 (28.6) | |
| T2 | 213 (65.7) | 20 (71.4) | |
| N stage positive | 64 (19.8) | 8 (28.6) | |
| N1 (N1a/N1b) | 54 (33/21) (84.4) | 6 (4/2) (75.0) | |
| N2 (N2a/N2b) | 10 (7/3) ) (15.6) | 2 (1/1) (25.0) | |
| Lymph node yield | 20 [15– 29] † | 17.5 [11.3 – 31.8] † | |
| **Lymph node yield ≥ 12** * | 290 (89.5) | 21 (75.0) | |
| Lymphovascular invasion | 38 (11.7) | 5 (17.9) | |
| Perineural invasion | 23 (7.1) | 1 (3.6) | |
| Differentiation |  |  | |
| Poor | 10 (3.1) | 3 (10.7) | |
| Moderate | 250 (77.2) | 22 (78.6) | |
| Well | 64 (19.8) | 3 (10.7) | |
| Follow-up (month) | 80.4 [51 – 109] † |  | |
| Total follow-up length | 83.0 [51 – 109] † | 64.0 [46 – 85] † | |
| Time to local recurrence |  | 27.0 [15.8 – 41.0] † | |
| Time to distant metastasis |  | 26.5 [12.9 – 47.6] † | |

*BMI*: Body Mass Index, *CEA*: Carcinoembryonic Antigen, *CRT*: chemoradiotherapy

*** *p* value** < 0.05

† Median [25 percentile – 75 percentile].

‡ Short-course radiotherapy: 500cGy*5days.
