# Supplementary material for: The risk factors of local recurrence and distant metastasis on pT1/T2N0 mid-low rectal cancer after total mesorectal excision
Source: World J Surg Oncol. 2021 Apr 13;19:116. doi: 10.1186/s12957-021-02223-4 (PMC8045195; doi:10.1186/s12957-021-02223-4)
Supplement: Supplementary file 2 — Additional file 2: Supplementary table 2 Patient characteristics for non-RT vs. RT [file 12957_2021_2223_MOESM2_ESM.docx]

**Supplementary table 2** Patient characteristics for non-RT vs. RT

| Variable | All 279 patients (% or [Q1 - Q3] †) | | |
| --- | --- | --- | --- |
|  | No Radiotherapy  (n=232, %) | | Radiotherapy +  (n=47, %) |
| Age | 63.6± 12.6 | 62.5 ± 11.5 | |
| BMI (kg/m2) | 24.2± 3.2 | 23.9 ± 3.4 | |
| Male Gender | 128 (55.2) | 25 (53.2) | |
| Family cancer history | 73 (31.5) | 14 (29.8) | |
| Pre-operative CEA (ng/mL) | 1.8 [1.1- 2.7] † | 1.9 [1.3 – 3.4] † | |
| Pre-operative CEA ≥ 5 | 30 (12.9) | 7 (14.9) | |
| Pre-operative Hemoglobin (g/dL) | 12.9 ± 1.9 | 12.8 ± 2.3 | |
| Pre-operative Albumin (g/dL) | 4.25 ± 0.38 | 4.16 ± 0.47 | |
| Distance from anal verge (cm) | 5.9 ± 1.8 | 5.7 ± 1.6 | |
| Distance from anal verge ≤ 5 | 97 (41.8) | 23 (48.9) | |
| Operation type |  |  | |
| Low anterior resection | 214 (92.2) | 44 (93.6) | |
| Abdomino-perineal resection | 16 (6.9) | 3 (6.4) | |
| Hartmann’s procedure | 2 (0.9) | 0 | |
| Adjuvant therapy | 3 (1.3) | 1 (2.1) | |
| Chemotherapy | 2 (0.9) | 1 (2.1) | |
| CRT | 1 (0.4) | 0 | |
| **Peri-OP colostomy/ileostomy*** | 124 (53.4) | 40 (85.1) | |
| Post-OP complication/morbidity | 57 (24.5) | 14 (29.8) | |
| Early | 38 (16.3) | 8 (17.0) | |
| Late | 31 (13.3) | 7 (14.9) | |
| Resection margin (cm) | 1.5 [0.8– 2.2] † | 1.3 [0.6 – 2.0] † | |
| Resection margin < 0.9 | 72 (31.0) | 16 (34.0) | |
| Resection margin < 1.4 | 108 (46.6) | 24 (51.1) | |
| Tumor diameter (cm) | 2.8 [2.0 – 4.0] † | 2.7 [2.0 – 3.5] † | |
| Tumor diameter (cm) ≥ 2.7 | 123 (53.2) | 27 (57.4) | |
| **T stage*** |  |  | |
| T1 | 94 (40.5) | 9 (19.1) | |
| T2 | 138 (59.2) | 38 (80.9) | |
| Lymph node yield | 20 [15– 29] † | 19 [13 – 26] † | |
| Lymph node yield ≥ 14 | 183 (78.9) | 35 (74.5) | |
| Lymphovascular invasion | 12 (5.2) | 1 (2.1) | |
| Perineural invasion | 10 (4.3) | 3 (6.4) | |
| **Differentiation*** |  |  | |
| Poor | 3 (1.3) | 2 (4.3) | |
| Moderate | 168 (72.4) | 41 (87.2) | |
| Well* | 61 (26.3) | 3 (6.4) | |

*BMI*: Body Mass Index, *CEA*: Carcinoembryonic Antigen, *RT*: radiotherapy, *CRT*: chemoradiotherapy.

*** *p* value** < 0.05

† Median [25 percentile – 75 percentile].

‡ Short-course radiotherapy: 500cGy*5days.
